# Weaning unit and prolonged mechanical ventilation after critical illness

**DOI:** 10.1186/2197-425X-3-S1-A158

**Published:** 2015-10-01

**Authors:** JA Rubio Mateo-Sidron, E Palma Gonzalez, J Rubio Quiñones, R Sierra Camerino, F Carmona Espinazo, F Fuentes Morillas

**Affiliations:** Hospital Infanta Cristina, ICU, Badajoz, Spain; Hospital Infanta Cristina, Badajoz, Spain; Hospital Puerta Del Mar, Cádiz, Spain

## Introduction

Patients requiring prolonged mechanical ventilation (PMV) and protracted and weaning after critical illness is rising. The consequences are higher Intensive Care Unit (ICU) costs and length of hospital stay. Weaning Unit (WU) with a multidisciplinary expert team may facilitate weaning and hospital discharge.

## Objectives

To compare risk factors and outcomes of patients who require tracheostomy, PMV and weaning after critical illness and are transferred to a WU or to a general ward (GW).

## Methods

We retrospectively reviewed medical records of tracheostomized and clinically stable ICU adults patients who required PMV (> 21 days) and weaning (> 7 days) and were transferred to a WU or a GW over a 8-year period (2007-2014) after critical illness. The study was carried out in two tertiary care university hospitals. Study variables were age, sex, APACHE II score, principal diagnosis, associated major procedures, length of stay in ICU and out in hospital, TCU and GW, Sabadell score, in-hospital mortality, types of tracheotomy procedure, decision to decannulate and discharge to home or long-care facilities.

## Results

In total 66 records of patients discharged from ICU were analysed. Two groups were defined: 1) WU (n= 26) and 2) GW (n= 40). Patients of WU group were older (60.88 ± 16.5 vs 55.4 ± 17.05 years) with higher APACHE II score (27.8 [CI: 24.3 to 31.3] vs 16.2 [CI: 14.1 to 18.3]), and had longer stay in ICU (45,5 [CI: 40.8 to 56.8] vs 19.5 [CI: 19.1 to 31.4] days; P < 0,001) and in ward (74.5 [CI: 63.7 to 115.8] vs 28 [CI: 21.7 to 36.3] days; P < 0,001) than those of GW group. Rates of nosocomial infections, vasoactives use, renal failure, blood transfusions were similar in both groups. in-hospital deaths, decannulation or discharge to home. More patients were transferred to long-care facilities from hospital without WU (4 [15.4%] vs 19 [47.5%] P < 0.001).

## Conclusions

Weaning Unit should be considered in hospital and ICU configuration for an integral assistance of critically ill patients with PMV and weaning.

## References

[1] Lone NI, Walsh TS (2011). Prolonged mechanical ventilation in critically ill patients: epidemiology, outcomes and modelling the potential cost consequences of establishing a regional weaning unit. Critical Care.

[2] Bigatello LM, Stelfox HT, Berra L (2007). Outcome of patients undergoing prolonged mechanical ventilation after critical illness. Crit Care Med.

